# Depressive symptoms, atherosclerotic burden and cerebral blood flow disturbances in a cohort of octogenarian men from a general population

**DOI:** 10.1186/1471-244X-13-347

**Published:** 2013-12-26

**Authors:** Arkadiusz Siennicki-Lantz, Lena André-Petersson, Per Wollmer, Sölve Elmståhl

**Affiliations:** 1 Division of Geriatric Medicine, Department of Health Sciences, Skane University Hospital, Jan Waldenströmsgata 35, CRC, Building 28, floor 13, SE-205 02 Malmö, Sweden; 2 Department of Clinical Sciences, Skåne University Hospital in Malmö, Malmö, Sweden; 3 Clinical Physiology and Nuclear Medicine Unit, Department of Clinical Sciences, Lund University, Lund, Sweden

**Keywords:** Vascular depression, Elderly, Subcortical, Cerebral blood flow, Ankle-brachial index

## Abstract

**Background:**

The aim of this study was to examine in elderly men a relationship between depressive symptoms, peripheral vascular disease and cerebral blood flow (CBF).

**Methods:**

Population-based cohort study started with an examination of 809 men at age 55, followed by the first (age 68ys) and second follow up (age 82ys). 128 survivors were examined at age 82 with ^99m^Tc-HMPAO-SPECT to estimate CBF, Zung-Self-Rating-Depression Scale (ZSDS), and Ankle-Brachial Index (ABI). Analysis was performed on men free from stroke and dementia which defined the final study population to 120 subjects.

**Results:**

ZSDS in the whole cohort ranged from 0.26 to 0.71 (reference 0.25-1.0). As the frequency of depressive symptoms was low, the case group (n = 31) was defined by ZSDS index above 75th percentile (≥0.48), comprising 9 subjects with *mild depression* (ZSDS 0.55-0.71) and 22 subjects at 88th percentile and above of the *normal range* (ZSDS index 0.48-0.54). Cases were more often current smokers at age 68 (44% vs. 24%; *p = .02*) and had lower systolic blood presure (SBP), lower social and physical activity, and suffered from fatigue, nausea, freezing and leg edema at age 82. Within the case group, ZSDS-index correlated negatively with CBF in subcortical area (r = -.42*), left and right thalamus (r = -.40*, r = -.46**), and right basal nuclei (r = -.35*). ZSDS-index correlated also with ABI at age 82 (right leg r = -.40*; left leg r = -.37*), and with Δ between ABI at age 82 and 68 (right r = -.36*; left r = -.46**). Despite decreasing SBP from age 68 to 82, adjusted multiple regression analysis showed in the case group that higher SBP determined CBF changes in the frontal and parietal areas, independently of ZSDS index, Δ ABI, and smoking.

**Conclusion:**

In this population-based cohort of octogenarian men free from stroke or dementia, a proportion of subjects with depressive symptoms was low. Still, men with borderline or mild depression scores had lower social and physical activity, persistent smoking habit, worse peripheral circulation in legs, and cerebral perfusion changes in basal nuclei, thalamus and subcortical white matter. Regional CBF decline could be partly mediated by higher SBP.

## Background

The pattern of depression in the elderly differs from that observed in a younger population. Several somatic symptoms, eg.: constipation, weight loss, restlessness, feeling of dependence, and poor self-perception of health, coexist with emotional symptoms and are included in Geriatric Depression Scale or Zung Self-Rating Depression Scale [1]. The concept of vascular depression comprised cerebrovascular disease as a predisposing, precipitating and perpetuating factor for depressive symptoms [2]. Even if risk of dementia is doubled in elderly depressed, there is still unclear whether depressive symptoms represent a risk factor for dementia or a reaction to early cognitive decline [3]. Depression–executive dysfunction syndrome is another variant of an affective diseases in elderly with observed impairment in frontolimbic and frontostriatal pathways and involve neocortex, amygdala or basal ganglia. The possible mechanism for these changes could be vascular or degenerative, or an accumulation of both factors [4]. Study results suggesting vascular impact in the elderly depression presented higher frequency of hypertension earlier in life, in those who later developed depression (72% vs 52%), and a very clear blood pressure decline in the preceding years [5]. Brain atrophy observed in younger elderly with hypertension and subsequent blood pressure decline predict poorer somatic health, cognition and shorter survival [6]. There is still a lack of cohort studies, based on non-selected elderly, studying a role of longitudinal vascular components in depressive symptoms.

The aim of this study was to examine, in a general population sample of 82-years-old men, a frequency of self-rated depressive symptoms and their association with peripheral vascular disease in legs, as well as with regional changes in cerebral blood flow.

## Methods

### Follow up of the cohort

**Figure 1:**
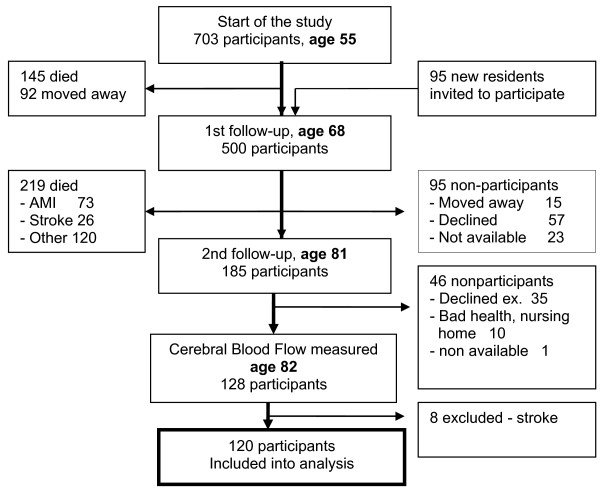
**Follow**-**up of the cohort**, **“Men born in 1914”.**

A prospective population study ”Men born in 1914” has been in progress since 1968 and included all men born in even months in 1914 in the city of Malmö, Sweden. Totally, 809 men were invited to and 703 men took part in the first health examination at age 55 (Figure 1). When they were 68 years old, 465 men in the cohort and additionally 95 new residents were invited to a first follow up. Five hundred of them agreed to participate. The second and last follow-up started when subjects reached 81–82 years. At that age, 281 men were found to be still alive. Of these, 185 agreed to take part (66%) in a clinical examination. In the following year, subjects were asked to participate in cerebral blood flow (CBF) examination. During that year, 10 subjects had died and 46 did not want to participate for other causes. Finally, our study population was defined to 128 subjects whose CBF was examined and all clinical data were available. Dementia and stroke were identified in eight subjects as described below. Depression in stroke is well defined, as well as biasing association between dementia and depression, wherefore those eight subjects were excluded from analysis. Thus, 120 men were finally included to the statistical analysis.

### Health examination

At the both follow ups, all study subjects were interview and examined by a doctor (at age 82 by a geriatrician). Medical history was also retrieved from spouses and/or relatives. Hypertension was defined according to WHO guidelines from 1986, i.e. systolic and diastolic brachial blood pressure ≥ 160 mmHg or ≥ 90 mmHg respectively, or medication for hypertension, as these guidelines were used for initiation of therapy at the time for both follow-ups. 15 questions considering social and physical activites were included into the questionnaire. Dementia diagnosis was classified by a geriatrician according to DSM IV. Mini Mental State Examination was performed in 125 men by a neuropsychologist and all but one subject had score ≥ 24 (Mean 28.3; SD 1.71). Information on incidents of stroke during whole follow up was registered during health examination, or retrieved from medical records and local stroke-registry. Stroke was defined according to ICD-9 classification codes 430.00-438.99. Two groups were defined concerning the smoking habit at age of 68: non-smokers together with former smokers, and current smokers.

### The zung self-rating depression scale

ZSDS scale was used to survey the depressive symptoms and was performed by a research psychologist. The scale consists of 20 items using four-point-grading system, ranging from “none or a little of the time” to “most of or all the time” [1], and is a sensitive tool to detect clinical depression in the elderly [7,8] The ZSDS does not give the severity of depression accurately but is a sensitive tool to detect clinical depression in the elderly, and the results are explained by cognitive deficits only to a lesser extent. ZSDS have high sensitivity (88%) at the recommended cut off points below, and the highest positive predictive value, 93%, and the greatest internal consistency. The ZSDS score has been calculated into the ZSDS index, derived by dividing the sum of the raw scores obtained on the 20 items by the maximum possible score of 80, and expressed as a decimal point. In previous studies, depression is described according to the cumulated score (index) as follows: 20–44 (0.25-0.55) Normal Range; 45–59 (0.56-0.74) Mildly Depressed; 60–69 (0.75-0.86) Moderately Depressed, 70 and above (0.88-1.0) Severely Depressed [7]. In the studied group ZSDS scores (indexes) ranged between 21p (0.26) and 57p (0.71), (Mean 34.5p; SD 6.0p). Since the frequency of depression in this populations based cohort occurred to be low, we chose for statistical analysis to divided our study group into quartiles. We defined subjects in the highest ZSDS quartile (75th percentile; index ≥0.48p) as a case group, contrary to the rest of the sample.

### Cerebral blood flow estimation

Cerebral blood flow has been estimated using single photon emission tomography (SPECT). An intravenous injection of 800 MBq 99mTc-HMPAO (Ceretec; Amersham Inc.) was given. The acquisition was performed during resting conditions on a triple headed gamma camera system (Siemens Multispect 3, Siemens, USA). Technical specifications and image processing have been described previously [9]. Regions of interest (ROI:s) were delineated separately in each hemisphere, in cortex of: frontal, parietal, and temporal lobes, both basal nuclei, both thalami and one ROI in subcortical white matter, bilaterally. The measured value in each ROI was quantified as a percentage of the mean cerebellar count ratio.

### Peripheral arterial circulation

Ankle blood pressure was estimated with the same manner at the ages of 68 and 82 years, by a technician at the department of Clinical Physiology, using standardized method, by placing a cuff around the ankle, and using a Doppler signal on the tibial posterior artery or dorsal foot artery to detect peripheral blood flow. The reference pressure in the arm was calculated using the strain gauge system. The ankle-brachial pressure index (ABI) was calculated for each leg by dividing the ankle systolic blood pressure by the highest individual upper arm systolic blood pressure value. ABI values greater than 1.3 was considered abnormal and subjects would be excluded. As a marker of progression of peripheral arterial disease, an arithmetic difference between ABI at age 82 and at age 68 has been calculated on each side and used in analyses (Table 1).

**Table 1:** Background data in subjects with mild depressive symptoms and in control group

**Background data**	**ZSDS index ≥ .48 n = 31**	**Controls n = 89**	***p***
*Medical history:*			
Mini mental state exam. (score)	28.4 (2.2)	28.4 (1.5)	ns.
Current smoker at age 82 (%)	15.6	18.2	ns.
Current smoker at age 68 (%)	43.8	23.9	.042
Alcohol consumption et least once last month, age 82 (%)	69.4	62.1	ns.
Alcohol consumption et least twice a week, age 68 (%)	28.1	29.5	ns.
Myocardial infarction (%)	6.5	8.0	ns.
Hypertension at age 82 (%)	40.5	55.7	ns.
Hypertension at age 68 (%)	18.8	55.7	.0004
Antihypertensive drugs at age 82 (%)	15.6	25.3	ns.
Fracture (%)	9.4	13.6	ns.
Cancer between age 68–82 (%)	12.5	11.4	ns.
Prostate disease (%)	40.6	34.1	ns.
*Clinical symptoms and signs:*			
Leg oedema (%)	15.6	1.1	.005
Dyspnoea at rest (%)	9.4	3.4	ns.
Back pain (%)	46.9	33.0	ns.
Joint pain (%)	30.0	33.0	ns.
Trouble passing urine (%)	15.6	6.8	ns.
Freezing (%)	40.6	20.5	.034
Tendency to emaciation (%)	9.4	5.7	ns.
Abdominal pain (%)	21.9	10.2	ns.
Fatigue (%)	53.1	28.7	.018
Nausea (%)	43.8	22.7	.038
*Social situation and social activities:*			
Education ≥10 years (%)	21.8	19.3	ns.
Widowed (%)	28.1	17.0	ns.
Living alone (%)	37.5	22.7	ns.
Do you cook for yourself ? (yes, %)	62.4	79.5	.025
Dissatisfied with level of activity (partly or very, %)	21.9	1.1	.0001
Feel stressed (yes, %)	31.2	8.0	.003
Ceased to exercise (yes, ?)	43.8	25.3	.008
Ceased hobbies (yes, ?)	40.6	16.1	.014
Poor social life (yes, %)	21.9	6.9	.032
Social contacts have changed (yes, %)	28.1	5.7	.003
How many people do you know? (≥10 persons, %)	28.1	52.9	.009
Would get help from someone if he was sick (no, %)	15.5	0.0	.001
As easy to manage money as before (yes, %)	93.8	97.7	ns.
Voted in the last general election (no, %)	12.5	1.1	.018
Can go fast 5 min? (no, %)	25.0	9.1	.033

ns. non-significant.

### Statistics

Summary values were expressed as mean ± standard deviation or as percentage if dichotomous. Comparing background data between cases and controls, Chi-square test/Fisher’s test was used for frequencies. The analyses of difference in CBF and ABI were made with either T-test for Independent Samples or Mann–Whitney *U* test. Correlation analyses between CBF or ABI and ZSDS index were performed using Pearson or Spearman correlation tests, depending on the data distribution. A General Linear Model was used to study an association between CBF in several brain areas (dependent variable) and ZSDS index, Systolic Blood Pressure, Δ Ankle Brachial Index left side - age 82-68ys., and non/previous smokers against active smokers at age 68, as independent variables in a fully adjusted model. A two tailed *P* value of less than 0.05 was considered statistically significant. All data analyses and statistical calculations have been performed using SPSS (SPSS Inc., Chicago, USA) data package. Local ethical committee had approved the study, and informed consent was obtained from all participants.

### Ethics

The study was approved by the local ethics committee at Lund University (LU 111–82). All subjects gave their informed consent.

## Results

Of the 128 subjects in the study population, eight suffered from a stroke during the preceding years and were excluded from the analysis. In the remaining 120 subjects, the 75th percentile of ZSDS index was estimated to 0.48. Thus, 31 subjects with ZSDZ index ≥0.48 were defined as case group, compared to 89 subjects with ZSDS index < 0.48 points. According to the qualitative description of symptom grades, our case group comprised 9 subjects with *mild depression* (ZSDS index 0.55-0.71) and 22 subjects at 88th percentile and above of the *normal range* (ZSDS index 0.48-0.54).

Case group was characterized by a double frequency of current smokers at age 68, i.e. 14 years before the depression survey, compared to the controls (44% vs. 24%; *p = .042*) (Table 1). In the studied cohort, ZSDS index in 35 current smokers was higher than in 85 non- and former smokers (0.46 ± 0.09 vs. 0.42 ± 0.06; *p = .02*). Systolic blood pressure was over 10 mmHg lower (151.8 ± 21.5vs. 162.5 ± 24.4*; p = .03*) in the case group compared to the controls, as previously reported [5], while frequency of hypertension was significantly lower at age 68, and slightly lower at age 82 (Table 1). Somatic symptoms differed between the groups, with significantly higher frequency of: fatigue, nausea, freezing, leg edema, in the case group, and slightly higher, if non-significant, of abdominal pain or dyspnea. The social situation differed slightly between the groups, with higher frequency of widowed and living alone among cases, without reaching significance level, but with a significantly lower social activity level, concerning hobbies, exercise, human contacts and voting. Study subjects experienced their activity level as dissatisfactory and had higher subjective feeling of stress (Table 1). Even if none of the subjects had severe handicap or were dependent on physical aids (unpublished data), the number of subjects in the case group who were not able to go fast for 5 minutes was nearly three times as high as in controls.

**Figure 2:**
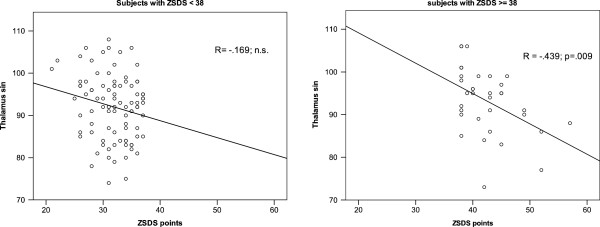
Correlation between ZSDS score and cerebral blood flow in left thalamic region assessed in octogenarian men with depressive symptoms (ZSDS index ≥ 0.48) and in controls.

There was no difference between the case and control group concerning CBF level or ABI-values (Table 2). Instead, an analysis within the groups was performed, showing in case group a negative correlation between ZSDS index with CBF in subcortical area, left and right thalamus, and basal nuclei (Table 3 and Figure 2). No such correlation was observed in the control group. ABI at age 68 did not predict ZSDSindex 14 years later, nevertheless, ABI estimated at age 82 correlated negatively with ZSDS index both in the case and the control group (Table 3), i.e. subjects with the lowest ABI had higher ZSDS index. The arithmetic difference in-between ABI-es measured at age 82 and 68 (ΔABI 82-68ys), especially in the left leg, was inversely associated with ZSDS index in the case group, i.e. an increase in the ZSDS index with decreasing ABI during the preceding 14 years.

**Table 2:** Distribution of cerebral blood flow and ankle brachial index (mean, SD) in subjects with mild depressive symptoms and in the control group

	**ZSDS index ≥ 0.48 n = 31**	**Controls n = 89**	***p***
*CBF, regions:*			
Frontal dx	81.0 (5.9)	80.4 (6.4)	ns.
Sin	80.6 (5.9)	80.0 (6.3)	ns.
Temporal dx	79.5 (5.0)	79.6 (5.5)	ns.
Sin	78.2 (5.1)	78.2 (5.4)	ns.
Paretal dx	81.9 (6.1)	80.7 (6.4)	ns.
Sin	80.5 (7.1)	80.0 (6.3)	ns.
Basal ganglia dx	93.6 (6.7)	92.1 (7.3)	ns.
Sin	92.3 (6.7)	90.8 (7.2)	ns.
Thalamus dx	93.7 (7.9)	92.5 (8.1)	ns.
Sin	93.0 (8.1)	92.0 (7.7)	ns.
Subcortical	65.5 (6.7)	63.2 (.9)	ns.
*Ankle brachial index:*			
Age 68, dx	1.15 (.138)	1.11 (.105)	ns.
Age 68, sin	1.11 (.144)	1.08 (.107)	ns.
Age 82, dx	.97 (.168)	.99 (.199)	ns.
Age 82, sin	1.01 (.183)	.96 (.185)	ns.
ABI-difference age 82–68, dx	-.16 (.136)	-.11 (.192)	ns.
ABI-difference age 82–68, sin	-.08 (.101)	-.12 (.168)	ns.

ns. non-significant.

**Table 3:** Coefficients of correlation (r) between ZSDS-index, Cerebral Blood Flow and ankle-brachial indexes, in case and control groups

	**ZSDS index ≥ 0.48, n = 31**		**Controls, n = 89**	
*CBF, regions:*	r	*p*	r	*p*
Frontal dx	-.11	ns.	-.09	ns.
Sin	-.12	ns.	-.04	ns.
Temporal dx	-.21	ns.	-.07	ns.
Sin	-.15	ns.	-.03	ns.
Parietal dx	-.29	ns.	-.04	ns.
Sin	-.21	ns.	-.01	ns.
Basal nuclei dx	-.35	.047	-.10	ns.
Sin	-.30	ns.	-.11	ns.
Thalamus dx	-.46	.008	-.15	ns.
Sin	-.40	.023	-.17	ns.
Subcortical	-.42	.014	-.12	ns.
*Ankle brachial index*				
Age 68, dx	-.15	ns.	.03	ns.
Age 68, sin	-.18	ns.	-.09	ns.
Age 82, dx	-.40	.02	-.21	.04
Age 82, sin	-.37	.03	-.24	.02
Difference 82–68, dx	-.36	.04	-.19	ns.
Difference 82–68, sin	-.46	.006	-.15	ns.

ns. non-significant.

Multiple linear regression analysis showed in a non-adjusted model a negative association between Cerebral Blood Flow in bilateral thalamic areas and subcortical white matter and ZSDS index (Table 4). In an adjusted model for systolic blood pressure, Δ left Ankle Brachial Index between ages 82-68ys., and non/previous smoking against active smoking, ZSDS index was associated with CBF only in right thalamus. Instead, higher systolic blood pressure independently determined decreased CBF in both frontal lobes and especially in both parietal areas (Table 4). Associations above were observed only in the case group.

**Table 4:** **Linear regression coefficients B(SE) calculated in the case group (n = 31) for the association between cerebral blood flow in several brain areas (dependent variable) and ZSDS index as independent variable in a non-adjusted model, and ZSDS index, systolic blood pressure,** Δ **ankle brachial index left side - age 82-68ys., and non/previous smokers against active smokers at age 68, as independent variables in a fully adjusted model**

	**Fr-R**	**Fr-L**	**Tem-R**	**Tem-L**	**Par-R**	**Par-L**	**BasG-R**	**BasG-L**	**Tha-R**	**Tha-L**	**Subc**
**Non adjusted model**											
ZSDS index	-20.2(18.8)	-18.0(18.8)	-25.1(15.9)	-12.4(16.4)	-29.9(19.1)	-20.2(22.1)	-39.5(20.6)	-28.9(21.5)	-58.5(23.1)*	-54.7(24.5)*	-41.9(20.4)*
**Adjusted model**											
ZSDS index	-23.6(20.1)	-21.5(19.7)	-17.8(17.0)	-11.3(18.1)	-26.3(18.1)	-19.3(21.3)	-39.4(22.7)	-20.2(23.5)	-54.8(25.8)*	-40.1(26.2)	-34.6(22.3)
SBP at 82	-.11(.05)*	-.12(.05)*	-.08(0.4)	-.08(.05)	-.17(.05)**	-.21(0.6)**	-1.0(22.7)	-.09(.06)	-.05(.07)	-.10(.07)	-.09(.06)
ΔABI82-68	-6.1(14.2)	-7.9(13.9)	-1.9(12.1)	-9.3(12.8)	-3.6(12.8)	-10.1(15.1)	.02(16.1)	12.4(16.6)	1.7(18.3)	8.3(18.5)	10.0(15.8)
Non-smoking	-1.8(2.6)	-1.5(2.6)	1.7(2.2)	1.3(2.3)	-.9(2.4)	-1.1(2.8)	-2.0(3.0)	-1.9(3.1)	.37(3.4)	1.4(3.4)	-1.8(2.9)

*p < .05 **p < .005.

*Abbreviations:Fr-R*: frontal right; *Fr-L*: frontal left; *Tem*: temporal; *Par*: parietal; *Occ*: occipital; *BasG*: basal ganglia; *Tha*: thalamus; *Subc*: subcortical.

## Discussion

In a presented study on a population of octogenarian men without previous stroke or dementia, we found low frequency of depression. In a chosen subgroup with depression scale scores from 75th percentile and above, i.e. with very mild depressive symptoms or being in an upper level of normal depression score range, cases were characterised by persistent smoking habit, symptoms of fatigue, nausea, freezing, leg edema, lower social activity level and lower gait speed, as well as worse peripheral circulation in legs. Although there was no a difference in CBF between cases and controls, increasing ZSDS index in the case group was associated with cerebral perfusion decline in basal nuclei, both thalami and subcortical white matter, and with worsening peripheral arterial circulation during the last 14 observation years. Vascular background of CBF abnormalities was also supported by an observed association between high systolic blood pressure and CBF decline in parietal and frontal areas, even if blood pressure decrement is more dominant in the elderly men with depressive symptoms [1].

Our study population represents non-demented, stroke free and mainly independent group of octogenarian men with generally good emotional level. The arbitrary borderline for depression on ZSDS score has been defined to 45 points (index 0.56) [7], which was found only in 9 patients of the 120 included in the statistical analysis. Therefore, the use of 75th percentile to define “cases” comprised subjects in the upper normal range of ZSDS score. Since our study is based on a populations-based sample, the frequency of moderate or severe depression was not expected to be high. In another scandinavian study on 85-years old population sample, the mean ZSDS score was 27.9p (SD 6.4), which is even lower than in our study [7]. In that study, a chosen ZSDS cut off for depression was 40p (index 0.50). When assessing vascular disease in our age group, one should be aware of the selective mortality in those subjects who died already before the first follow-up at age 68, or between follow-ups, due to early hypertension, metabolic syndrome, intensive smoking and advanced atherosclerosis. It emerged survivors whose atherosclerotic burden has accelerated mainly in the last life-decade [10]. These survivors had also been less exposed to vascular risk factors than those who declined to take part in the last follow up, or had been institutionalised. A choice of ABI as a marker of vascular health is based on the observations of ABI reflecting risk for future stroke, coronary disease, death, cognitive decline and dementia [11-15]. A relation between Ankle-Brachial index and apathy has been newly described [16]. In advanced peripheral disease, leg-symptoms were associated with anxiety, depression, and anhedonia [17].

Even if the subcortical localisation and a grade of CBF decline were associated with ZSDS index in a non-adjusted model, the adjustment for vascular risk factors influencing depression independently supported a vascular rather than a degenerative background of a perfusion decline. Previously introduced concept of vascular depression [2] comprised also depressive symptoms related to stroke. In this cohort, with stroke-induced depression excluded, a formerly proposed term of subcortical ischemic depression could be used to describe the depression mechanism and aetiology [18,19]. In earlier studies, ageing non-demented patients with major depression and forgetfulness showed reduced CBF in lateral frontal, left thalamus and bilateral medial frontal areas [20]. In other in-clinic patients with major depression, over 50% have been defined as having a subcortical ischemic depression. The awareness that subcortical ischemic depression is a risk factor for higher mortality, disability and dementia, indicate that even very moderately depressive symptoms in men with generally good health should be treated as an early warning signal for functional decline and fast somatic deterioration. A differing profile of symptoms due to subcortical ischemic changes, described as “disconnection syndrome”, including psychomotor retardation, difficulty at work, apathy, lack of insight, and executive dysfunction [21,22], should be identified by general practitioners and geriatricians already in an early faze, as these symptoms are seldom an incentive for these men to visit a doctor. An opposite approach should also be recommended, with basic psychiatric/emotional assessment in elderly with high vascular load or signs of white matter lesions on brain imaging. In the correlations analysis we could observe a trend in negative correlation coefficients in all regions of the case group, pointing that subcortical grey and white matter changes could either influence the connectivity, expressed on CBF as retrograde blood flow decrement, or that ZSDS scale expresses a general brain atrophy or diminished perfusion through lobar arterioles as well. The significant difference in social activities between cases and controls, despite normal MMSE levels, and independence in ADL, could reflect early changes in cerebral function expressed by executive dysfunction.

In the regression analysis, the associations between ZSDS index and CBF after adjustment for vascular risks were reduced to right thalamus, which maybe explained that not only vascular but also degenerative processes could answer for subcortically mediated elderly depression. Another result worth attention is an association between systolic blood pressure and CBF in both frontal and especially in both parietal areas, independently of ZSDS score and other vascular risk factors. It could mean that CBF changes in a large part of the brain in early depression could be at least in some subjects caused by a high blood pressure. This result is surprising, since we previously have shown that a grade of depression in this cohort is related to the blood pressure decline from age 68 to age 82, especially in those who were hypertensive at age 68, thereafter reached an inflection point in blood pressure trajectories, followed by a pressure decline until age 82 [5]. We explained it with a possible heart failure, frailty or dysautonomia. In this analysis, it occurred that CBF changes in the case group are observed in those men whose blood pressure increased or who have had a persisting hypertension, probably causing silent cerebral damage in frontal and parietal areas. It could also mean that mild depressive symptoms in a general population of the elderly men are multifaceted, may have different mechanisms, and could not exclusively be explained by cerebrovascular disease.

The strengths of this study are: a well-examined cohort sample, lack of age and gender bias and a good quality of longitudinal follow-up. The medical examination was performed by an experienced geriatrician and the psychological interview by a clinical neuropsychologist. Among the limitations of this study, we should include a possible underrepresentation of institutionalized and non-native-Swedish elderly. Elderly with cognitive decline could also be more likely to decline participation in the last follow-up, and elderly men with depressive symptoms might be less inclined to answer openly on the questions included in the ZSDS. There are also epidemiological aspects in assessing older adults with questionnaires and interviews, e.g. difficulty remembering symptoms. We did not use criteria of depression for assessing cohort subjects, except ZSDS scoring, which could result in a restricted validity and specificity.

## Conclusion

In this population-based cohort of octogenarian men free from stroke or dementia, a proportion of subjects with depressive symptoms evaluated by Zung Self-Rating Depression Scale was low. Even so, men with borderline or mild depression scores had lower social and physical activity, persistent smoking habit, worse peripheral circulation in legs and cerebral perfusion changes in basal nuclei, thalamus and subcortical white matter. In these men, cerebral perfusion decline in parietal and frontal lobes was associated with higher systolic blood pressure, even if blood pressure decrement was more frequent over time than in non-depressed subjects.

## Competing interests

Authors declare that they have no financial or non-financial competing interests.

## Authors’ contributions

ASL was involved in data analysis and manuscript preparation. LAP carried out the neuropsychological analysis and supervised ZSDS estimation. PW carried out the cerebral blood flow estimation. SE was involved with study design, data collection and manuscript preparation. All authors read and approved the final manuscript.

## Pre-publication history

The pre-publication history for this paper can be accessed here:

http://www.biomedcentral.com/1471-244X/13/347/prepub
